# Multiple Alignment of Promoter Sequences from the *Arabidopsis thaliana* L. Genome

**DOI:** 10.3390/genes12020135

**Published:** 2021-01-21

**Authors:** Eugene V. Korotkov, Yulia M. Suvorova, Dmitrii O. Kostenko, Maria A. Korotkova

**Affiliations:** 1 Institute of Bioengineering, Research Center of Biotechnology of the Russian Academy of Sciences, Bld.2, 33 Leninsky Ave., 119071 Moscow, Russia; suvurovay@gmail.com; 2 National Research Nuclear University MEPhI (Moscow Engineering Physics Institute), 31 Kashirskoye Shosse, 115409 Moscow, Russia; dk0stenko@yandex.ru (D.O.K.); bioinf@rambler.ru (M.A.K.)

**Keywords:** multiple sequence alignment, promoter, dynamic programming, genetic algorithm

## Abstract

In this study, we developed a new mathematical method for performing multiple alignment of highly divergent sequences (MAHDS), i.e., sequences that have on average more than 2.5 substitutions per position (*x*). We generated sets of artificial DNA sequences with *x* ranging from 0 to 4.4 and applied MAHDS as well as currently used multiple sequence alignment algorithms, including ClustalW, MAFFT, T-Coffee, Kalign, and Muscle to these sets. The results indicated that most of the existing methods could produce statistically significant alignments only for the sets with *x* < 2.5, whereas MAHDS could operate on sequences with *x* = 4.4. We also used MAHDS to analyze a set of promoter sequences from the *Arabidopsis thaliana* genome and discovered many conserved regions upstream of the transcription initiation site (from −499 to +1 bp); a part of the downstream region (from +1 to +70 bp) also significantly contributed to the obtained alignments. The possibilities of applying the newly developed method for the identification of promoter sequences in any genome are discussed. A server for multiple alignment of nucleotide sequences has been created.

## 1. Introduction

Multiple sequence alignment (MSA) is one of the central tasks of bioinformatics. Significant efforts have been made to develop tools for MSA [1,2,3], which often include dynamic programming, progressive alignment, iterative methods, hidden Markov models (HMMs), and genetic algorithms [4,5]. The direct use of dynamic programming is hardly possible for the alignment of a large number of sequences (more than 10), since it is an NP-complete problem [6] and the calculations require considerable time. Therefore, some heuristic solutions have been developed, which are based on using the objective function for assessing the power of MSA and an optimization procedure. As a result, the quality of the constructed MSA is improved [2].

The progressive alignment is the most popular MSA algorithm. It includes pairwise comparison of *N* sequences, calculation of a matrix of distances between the sequences, construction of a matrix-based guide tree, and, finally, progressive MSA. Often, an optimization procedure is applied to the created progressive alignment to improve the final result and eliminate unnecessary deletions and insertions (indels). The sum of the similarity functions of pairwise alignments, which may be used as an objective function, can be obtained by projecting the constructed MSA on two-dimensional planes [7,8], resulting overall in *N*(*N*−1)/2 of pairwise alignments. Alternative target functions such as consensus [9], entropy [10], or circular sum [11] are also used. However, in most cases, progressive alignment cannot create the optimal MSA since the errors obtained at any stage of the procedure are accumulated at the final alignment. To minimize such errors, an optimization procedure that utilizes various mathematical algorithms is used. The most popular programs based on the progressive alignment are Clustal [12,13], MAFFT [14,15], and T-Coffee [16,17].

Iterative methods are another popular approach to construct MSA as they allow reducing the errors inherent in progressive alignment. When a new sequence is added to the MSA, the total alignment is recalculated, which is in contrast to progressive alignment when each originally calculated paired alignment is final. As a result, the error rate is reduced and the objective function is optimized. The most popular programs that employ iterative procedures are Muscle [18], PRRN [19], and CHAOS/DIALIGN [20].

HMMs, which are also used to construct MSA, consider the probabilities of all possible states, i.e., match, insertion, or deletion, for each character [21,22]. Although the Markov models, including the hidden ones, work, in fact, as a “replicating” method, they may improve the calculation speed. In the process, an initial MSA is constructed and used to determine the HMM parameters, which are then improved through iteration. Therefore, the application of HMMs to MSA is accompanied by several other optimization procedures and mathematical methods, often of an iterative nature, such as the Baum-Welch algorithm [10], simulation of annealing [23], gradient descent [24], etc. After optimization, an optimal Markov model is created and all sequences are aligned with it using the Viterbi algorithm [10] to find the best similarity between each sequence and the HMM.

The described approaches construct a statistically significant guide tree for progressive alignment based on pairwise alignments between sequences. However, in the case of highly divergent sequences, it is often not possible to find a statistically significant “germ” or common “words”. Therefore, it is currently extremely difficult or impossible to compute the MSA of highly divergent sequences.

In the case of highly divergent sequences, when each sequence pair has more than 2.5 substitutions per position (*x*), MSA becomes statistically significant only for a relatively large sample containing more than 10 sequences. MSA can be built using the *N*-dimensional dynamic programming for all the analyzed sequences, but it requires significant computational resources and is currently impossible to implement, suggesting that the available MSA approaches need improvement in this direction.

To address this problem, we have developed a new mathematical method for computing the multiple alignment of highly divergent sequences (MAHDS), which is partially based on previously described tandem repeat search algorithms [25,26]. Here, we upgraded such an algorithm and dynamic programming to compute the MSA by taking into account the correlation of adjacent characters. We have also developed a website for the calculation of a multiple alignment for very distantly related sequences using this method (http://victoria.biengi.ac.ru/mahds/main). The advantage of MAHDS is that it can produce a statistically significant alignment of a set of divergent nucleotide sequences for which any pairwise alignment does not reach sufficient statistical significance.

The analysis of the performance of currently available MSA programs, including ClustalW [27], Clustal Omega [28], T-Coffee [17], Kalign [29], MAFFT [14], and Muscle [18] in aligning nucleotide sequences depending on the degree of their evolutionary divergence (*x*) revealed that they are effective at *x* < 2.5. In comparison, the MAHDS program can construct a statistically significant MSA with *x* up to 3.7 for the number of sequences up to 100 and if the sequence set increases to 500, the statistically significant *x* limit is increased to 4.4. MAHDS was used to align promoter sequences from the *Arabidopsis thaliana* L. genome (from −499 bp upstream to +100 bp downstream of the transcription start site) and revealed that many upstream regions (from −499 to +1 bp) are highly conserved. A significant conservation was also observed in the regions from +1 to +70 bp. Furthermore, MAHDS provided identification of 25 promoter classes in the *A. thaliana* genome. The possibility of using the developed mathematical method and the calculated multiple alignment to identify promoter sequences in different genomes is discussed.

## 2. Methods and Algorithms

### 2.1. General Description of the MAHDS Algorithm

The main idea behind the MAHDS method is the construction of the optimal image rather than direct calculation of the MSA. Here, we used position-weight matrix (PWM) as such an image. For example, we have a set of sequences *sq*(1), *sq*(2),…, *sq*(*N*), which we combine into one sequence *S* of length *L* and then determine the best alignment between *S* and PWM that was originally calculated based on random sequence alignment and has the dimension of 4 × *L*/*N*. Then, sequence *S* is aligned against PWM using the Needelman-Wunsch algorithm [30] (for an example, see publication [31]) and the similarity between *S* and PWM is measured based on *F*(*L*,*L*) calculated by two-dimensional dynamic programming at the point (*L*, *L*). In the two-dimensional matrix *F* with the size *L* × *L*, sequence *S* is plotted in the X axis and the PWM, multiplied *N* times, in the Y axis. As a result, sequence *S* will be compared by dynamic programming with sequence *S*_1_, which contains the column numbers of the PWM matrix treated as characters and looks as 1, 2,…, *L*/*N*, 1, 2,…, *L*/*N*,…. We also created *S*_2_, which is a randomly shuffled sequence *S*. 

The purpose is to determine PWM with the greatest *F*(*L*,*L*), which then will be referred to as the best PWM. Using the created two-dimensional alignment, we can easily reconstruct the multiple alignment of the initial sequences. Therefore, MAHDS computes MSA by calculating *F*(*L*,*L*), finding the best PWM, and aligning sequence *S* to PWM.

**Figure 1:**
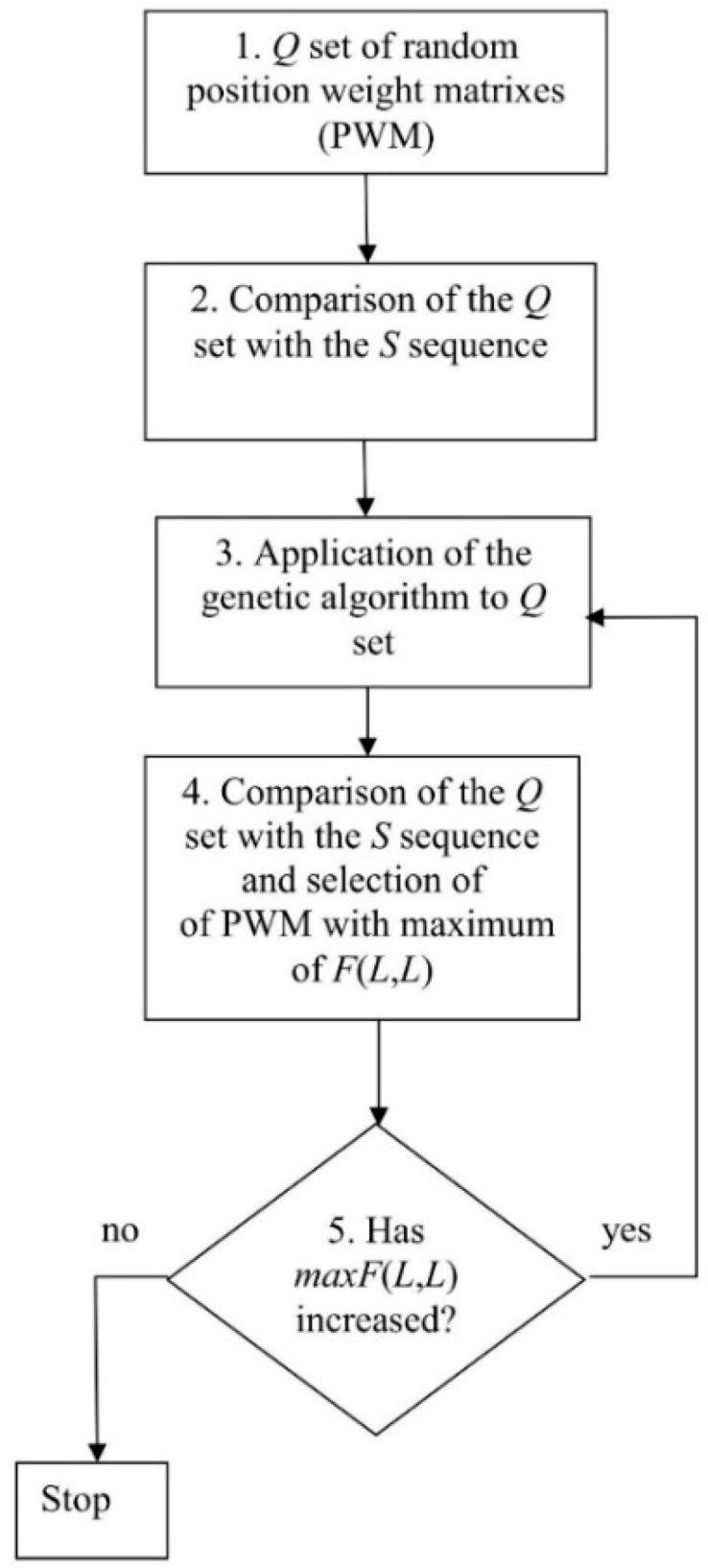
Algorithm for calculating the best position-weight matrix (PWM).

As it is extremely unlikely to obtain a good approximation of the optimal MSA from the first random PWM, we determined the best PWM using an optimization procedure. For this, we generated a set of random PWMs (*Q*) (Figure 1, point 1), which contained *n*_1_ random matrices, aligned matrix number *i* from set *Q* with sequence *S*, and calculated the corresponding *F*(*L*,*L*) value in position *i* of vector *V*(*i*) (*i* = 1,…, *n*_1_) (Figure 1, point 2). Then, vector *V*(*i*) was sorted in the ascending order and the matrices from set *Q* were arranged in accordance with the position of the corresponding value in *V*(*i*). 

Next, the genetic algorithm was applied to introduce random mutations into the matrices from *Q* and create hybrids (descendants) (Figure 1, point 3). The matrix corresponding to value *V*(1) (the smallest *F*(*L*, *L*)) was excluded from set *Q* and replaced with the descendant. Then, the values of vector *V*(*i*) were recalculated and *V*(*i*) re-sorted. The resultant matrix corresponded to the largest *F*(*L*, *L*) contained in *V(L)* and was denoted as *maxF*(*L*, *L*) (Figure 1, point 2). If the *maxF*(*L*, *L*) value decreased, then the process of matrix optimization was considered complete, if not—it was repeated. As a result, we obtained *maxF*(*L*, *L*), two-dimensional alignment of sequences *S* and *S*_1_, and the best PWM (Figure 1). The steps of this algorithm are described in detail below.

### 2.2. Creation of the Set of Random PWMs

We used random sequences to obtain a set of random PWMs (*Q*; Figure 1, point 1), which consisted of *L*/*N* columns and 16 rows. For this, we generated a random nucleotide sequence *S*_2_ of length *L* with equal base probability (0.25), transformed it into a numeric sequence (where bases a, t, c, and g were coded as 1, 2, 3, and 4, respectively), and compared it with sequence *S*_1_ of the same length *L* containing *N* repeated sequences (1, 2, …, *L*/*N*). Then, we filled frequency matrix *M*(*L*/*N*, 16) as:

*M*(*s*_1_(*i*), *s*_2_(*i* − 1) + 4(*s*_2_(*i*) − 1)) = *M*(*s*_1_(*i*), *s*_2_(*i* − 1) + 4(*s*_2_(*i*) − 1)) + 1 (1)

where *i* ranges from 2 to *L*. 

**Figure 2:**
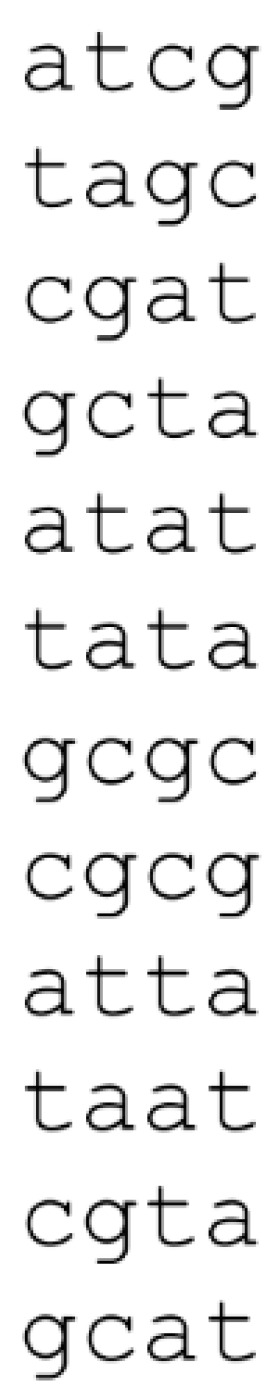
Multiple sequence alignment (MSA) in the case of correlations between neighboring bases.

This approach of matrix construction takes into account two effects in MSA [32]: The frequency of nucleotides at each position and the correlation of nucleotides in neighboring positions (*i* and *i* − 1), which allows multiple alignment of highly diverged sequences where base frequencies in each MSA position may not differ from those in sequence *S*, while maintaining the correlation of neighboring nucleotides. An example is shown in Figure 2, where short sequences were used for convenience. However, all conclusions are applicable to sequences of any length. Let us consider the following 4-nt sequences: Atcg, tagc, cgat, gcta, atat, tata, gcgc, cgcg, atta, taat, cgta, and gcat, which were constructed with dinucleotides at, ta, cg, and gc at positions 1–2 and 3–4. As a result, in each position of the MSA shown in Figure 2, the nucleotide frequency is 0.25, but nucleotide pairs 1–2 and 3–4 could be only at, ta, cg, or gc. The probability that the first and second columns contain only at, ta, cg, or gc is 0.0625 × 4 = 0.25 and the number of lines in the alignment is 12. Then, the expected number of each nucleotide pair is: 12 × 0.25 = 3. If a normal approximation for the binomial distribution is used, then the variance is 12 × 0.25 × (1 − 0.25) = 2.25. In total, the first two columns contain 12 pairs (at, ta, cg, or gc). Then, the normal distribution argument for this event is $(12-3)/\sqrt{2.25}$ = 6 and the probability due to purely random factors is less than 10^−6^. Given that this value is the same for columns 3 and 4, the probability of accidentally creating the alignment shown in Figure 2 is less than 10^−12^, which means that the individual columns in Figure 2 appear to be random, but there is a strong correlation between the adjacent bases.

Then, we calculated PWM matrix *W*(*L*/*N*, 16) using *M*(*L*/*N*, 16):

(2) $$W\left(i,j\right)=\frac{M\left(i,j\right)-Lp(i,j)}{\sqrt{Lp(i,j)(1-p(i,j))}}$$

where $p\left(i,j\right)=x\left(i\right)y\left(j\right)/L^{2}$, $x(i)=\sum_{j=1}^{4}M(i,j)$, $y(j)=\sum_{i=1}^{3}M(i,j)$, and $L=\sum_{i=1}^{3}\sum_{j=1}^{4}M(i,j)$. Then, matrix *W*(*i*,*j*) was transformed to obtain the given *R*^2^ and *K_d_*, calculated using the following formulas:

(3) $$R^{2}=\sum_{i=1}^{N/L}\sum_{j=1}^{16}w{\left(i,j\right)}^{2}$$

(4) $$K_{d}=\sum_{i=1}^{N/L}\sum_{k=1}^{16}w(i,k)p_{1}(i)p_{2}(k)$$

Here, *p*_1_(*i*) is the probability of symbols in *S*_1_, which is *N*/*L* for any *i*; $p_{{}_{2}}(k)=p(l)p(m)$, where *p*(*l*) and *p*(*m*) are the probabilities of the *l* or *m* type nucleotides in *S* (*l,m* ∈ {a,t,c,g}); *p*(*l*) *= q*(*l*)*/L*, where *q*(*l*) is the number of *l* type nucleotides in *S*; and *L* is the length of *S*. For all the calculations, we used *R*_0_ = 110*L*/*N* and *K*_0_ = −1.8 [32]. The matrix transformation procedure is described in detail in [25] and an example for the original matrix of five columns and *R*^2^ = 155 is shown in Table 1 (a small matrix is used to fit in full in Table 1). The transformed matrix has *R*^2^ = 2000 and *K_d_* =−1.5.

It is necessary to transform matrix *W* so that the distribution function for *F*(*L*, *L*) is similar for different *W* matrices. Therefore, the same *R*^2^ and *K_d_* values should be maintained. The optimal cost for an insertion or deletion (*del*, formula 5) depends on *R*^2^ and the average value of *F*(*L*, *L*) for a random sequence *S* depends on *K_d_*. The distribution function *F*(*L*, *L*) can be obtained by the shuffling of sequence *S* into 1000 random sequences denoted as set *SR* and calculating *F*(*L*, *L*) of matrix *W* for each sequence of the set. If *R*^2^ and *K_d_* for different transformed *W* matrices are the same, then *F*(*L*, *L*) distributions will be equal or similar [25], which is the objective of matrix transformation.

After obtaining one transformed PWM (*WT*) for set *Q*, we shuffled sequence *S* and repeated the calculations using Formulas (1) and (2). As a result, about 10^6^ PWMs for set *Q* were selected and then filtered to leave only those that uniformly fill the Euclidean space with dimension 16 *L*/*N*. We considered each matrix as a point in this space and calculated the Euclidean distance *D* between all matrices. We selected the threshold *D*_0_ so that set *Q* contained less than 10^3^ matrices. Any two matrices with *D* < *D*_0_ were excluded from the set. We denoted the number of matrices in set *Q* as *n*_1_.

### 2.3. Comparison of Set Q with Sequence S Using Dynamic Programming

**Figure 3:**
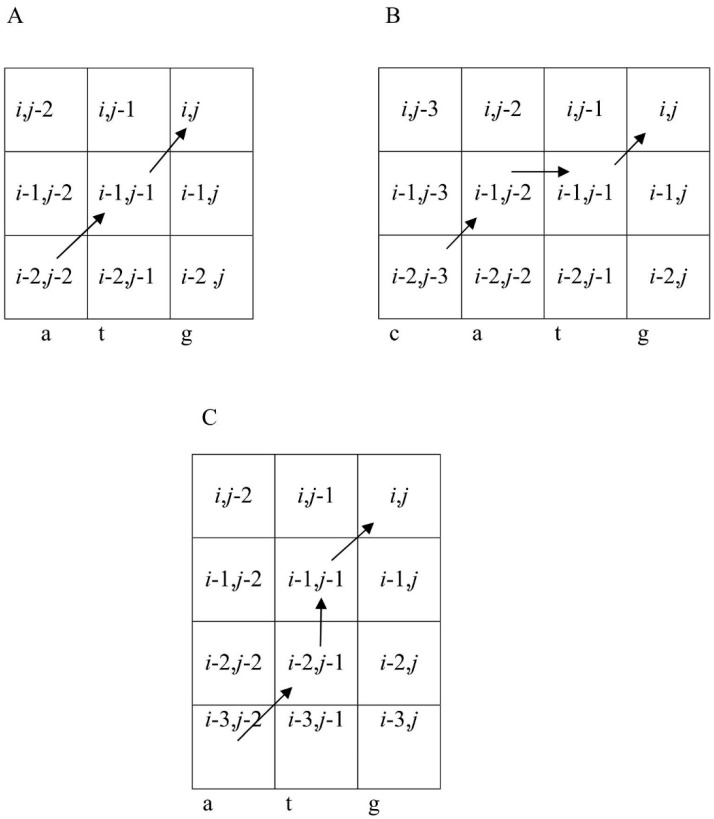
Three variants of the path from (*i* − 1, *j* − 1) to (*i*, *j*) to calculate *F*(*i*, *j*) using Formula (5) when *t* < 3. (**A**) The transition to (*i* − 1, *j* − 1) is from (*i* − 2, *j* − 2). Then, the previous position in the alignment is (*i* − 1, *j* − 1), which corresponds to *t* = 1. In this case, a pair of bases *s*(*j* − 1) = t and *s*(*j*) = g is chosen in sequence *S* and *k* = *j* − 1, whereas *s*(*k*) = *s*(*j* − 1) = t. Then, *n* = *s*(*k*) + 4(*s*(*j*) − 1)) = *s*(*j* − 1) + 4(*s*(*j*) − 1)) = 2 + 4 × (4 − 1) = 14, which means that we are using *W*(*s*_1_(*i*),14). (**B**) The transition to (*i* − 1, *j* − 1) is from (*i* − 1, *j* − 2) and that to (*i* − 1, *j* − 2) is from (*i* − 2, *j* − 3). In this case, *t* = 2, *k* = *j* − 2, and *s*(*k*) = *s*(*j* − 2) = 2, *n* = (*k*) + 4(*s*(*j*) − 1)) = s(*j* − 2) + 4(*s*(*j*) − 1)) = 1 + 4 × (4 − 1) = 13, and we use *W*(*s*_1_(*i*),13). (**C**) The transition to (*i* − 1, *j* − 1) is from (*i* − 2, *j* − 1) and that to (*i* − 2, *j* − 1) is from (*i*-3, *j* − 2). In this case, *t* = 2 and one symbol is deleted from the sequence. Then, we do not take into account the base correlation in sequence *S* and use matrix *W*_1_(*s*_1_(*i*),*s*(*j*)) and *n* = *s*(*j*) = 4.

Then, we aligned sequence *S* with each of the matrices from set *Q* using the global alignment algorithm. The *F* value was calculated as:

(5) $$F(i,j)=\max\left\{\begin{array}{l} F(i-1,j-1)+W(s_{1}(i),n)\\ F(i,j-1)-del\\ F(i-1,j)-del \end{array}\right\}$$

where *n* = *s*(*k*) + 4(*s*(*j*) − 1)), *i* and *j* each ranges from 2 to *L*, and *s*(*j*) and *s*_1_(*i*) are elements of sequences *S* and *S*_1_, respectively. Parameter *n* reflects the fact that in matrix *W,* dinucleotides were taken into account. To determine *n*, the previous position (*k*), already included in the alignment, should be defined. It was calculated from the created transitions in matrix *F*, depending on the previous base in *S* and used to obtain *W*(*s*_1_(*i*),*n*). If the previous base of sequence *S* is *s*(*j* − *t*), then *k* = *j* − *t* and *n* = *s*(*j* − *t*) + (*s*(*j*) − 1) × 4. Three cases should be considered. In the first, *t* = 1 corresponds to the movement along the main diagonal of matrix *F* and there is no deletion in sequence *S* in the alignment (Figure 3A). In the second, *t* > 1 corresponds to a deletion of *t* − 1 bases in sequence *S* (illustrated in Figure 3B for *t* = 2). Finally, deletions may occur simultaneously in both sequences *S* and *S*_1_, which correspond to deletions of columns in matrix *W*. If the previous symbol in sequence *S*_1_ has the number *i* − 1, then there is no deletion, but if this number is *i* − *t* (*t* > 1), then there is a deletion of *t* − 1 bases in sequence *S*_1_. For these transitions, we did not consider the correlations of adjacent bases and took *n* = *s*(*j*). Rather than matrix *W*(*s*_1_(*i*),*n*), we used matrix *W*_1_(*s*_1_(*i*),*s*(*j*)):

(6) $$W_{1}(s_{1}(i),s(j))=0.25\sum_{x=1,4}^{}W(s_{1}(i),x+(s(j)-1)*4)$$

In this case, the correlation of adjacent bases is not considered, which is quite acceptable when the number of deletions is relatively small (illustrated in Figure 3C for *t* = 2).

The zero row and column of matrix *F* were filled with negative numbers, *F*(0, *j*) and *F*(*i*, 0) were 0 for *i* and *j* ranging from 1 to *L*, respectively, and *F*(0, 0), *F*(1, 0), …, *F*(2, 0) were also equal to 0. Matrix *E*(*x*, *n*) was used to define the first column and row of matrix *F*. The insertion/deletion penalty value (*del* = 25.0) was selected based on our earlier work [25]. The reverse transition matrix was filled along with matrix *F*. Therefore, we aligned sequences *S*_1_ and *S* using the reverse transition matrix and determined *F*(*L*, *L*). The alignment of *S*_1_ and *S* was obtained for all matrices from the *Q* set. As a result, vector *V*(*i*) (*i* = 1, …, *n*_1_) contained *F*(*L*, *L*) for each matrix.

### 2.4. Application of the Genetic Algorithm to the Q Set

To optimize matrices from the *Q* set, we used a genetic algorithm described in our previous study [25]. The aim was to change each PWM from the *Q* set to maximize *F*(*L*, *L)*, which was considered an objective function. *F*(*L*, *L*) for each matrix was put into vector *V*(*i*) (*i* = 1, 2, …, *n*_1_), which was sorted in the ascending order from *V*(1) (the minimum) to *V*(*n*_1_) (the maximum) and the matrices in the *Q* set were arranged accordingly. Then, two matrices were randomly selected with the probability of choosing a matrix, which increased with the increase of *i* from 1 to *n*_1_, and the two matrices were used to create a “descendant”, for which any element of the first matrix was selected with an equal probability. Then, rectangles were randomly selected to the right and left above and below the selected element in the first matrix with the probability of 0.25 and the elements within the rectangle were moved from the first to the second matrix to create a descendant, which replaced the PWM with *V*(1).

Then, we introduced mutations in 10% of the randomly selected matrices from the *Q* set. To do this, a randomly selected element of the matrix was changed to a random value in the range from −10.0 to +10.0. Usually, less than 10^4^ cycles were required to achieve the moment when *V*(*n*_1_) did not increase, i.e., to reach the maximum designated as *maxV*(*n*_1_). However, in rare cases, more than 10^5^ cycles were performed. At the output of the algorithm (Figure 1), we obtained *maxV*(*n*_1_), two-dimensional alignment of sequences *S*_1_ and *S*, and matrix *maxW*, which were used to compute the alignment.

### 2.5. Calculation of Statistical Significance for maxV(n_1_)

We used the Monte Carlo method to estimate the statistical significance of *maxV*(*n*_1_). Sequence *S* was randomly shuffled to obtain 200 random sequences. Then, matrix *maxW* was included in the *Q* set described in Section 2.2, which allowed taking into account the effectiveness of *maxW* alignment with random sequences. Then, each of these sequences were treated as described in Section 2.2, Section 2.3, Section 2.4 and *maxW* was calculated for each, producing 200 *maxV*(*n*_1_). Then, the mean $\bar{maxV(n_{1})}$ and variance $\sqrt{D(maxV(n_{1}))}$ were calculated and used to compute *Z*:

(7) $$Z=\frac{maxV(n_{1})-\bar{maxV(n_{1})}}{\sqrt{D(maxV(n_{1}))}}$$

where *maxV*(*n*_1_) was calculated for sequences *S*_1_ and *S* in Section 2.4. 

*Z* was obtained for each MSA and the average Z value for random *S* sequences was estimated according to Formula (7) after each sequence had been subjected to the procedures described in Section 2.2, Section 2.3, Section 2.4 and here. As a result, the mean *Z* = 1.8, and we can assume that the MSA is non-random at Z > 6.0. 

### 2.6. MSA Construction

The MSA was computed using the two-dimensional alignment of sequences *S*_1_ and *S*. Each position in sequence *S*_1_ corresponded to a column in the MSA. Any insertion in the two-dimensional alignment (opposite to which there was a gap in sequence *S*_1_) resulted in an additional column in the MSA.

### 2.7. Comparison of Various MSA Methods

The algorithm shown in Figure 1 can also be applied to determine the statistical significance of MSAs created by other algorithms. Let us denote the MSA as *A*, the length of each sequence in *A* as *K*, and the number of sequences as *N*. All sequences from *A* are linked to produce sequence *S_3_* of length *L* = *KN*. Then, the PWM is calculated for *A* using Formula (2), transformed using Formulas (3) and (4), and applied to create the two-dimensional alignment for sequence *S*_3_ using Formulas (5) and (6) and to calculate *F*(*L*, *L*). The statistical significance of *A* is then computed according to Formula (7).

However, the columns that have a sum of elements < *N*/2 should be excluded from *A* to eliminate redundant deletions in the calculation of *F*(*L*, *L*), whereas those with the sum > *N*/2 cannot be excluded since it would lead to an excessive number of insertions. Consequently, the number of columns became *K′* ≤ *K*, resulting in a new alignment *A′* (*K′* is the length of each sequence in *A′*). To construct the PWM using *A′*, frequency matrix *M*(*K′*, 16) was first calculated using Formula (1) and then the PWM (designated as *W_A_*’) was calculated using Formula (2). Formulas (3) and (4) were applied to transform the resulting matrix and obtain matrix *WT_A_*_’_, which was used to calculate *F*(*L*, *L*) (*L* = *K′N*) based on *A*’. For this, the sequence from *A*’ was merged with sequence *S*_4_ with all the spaces preserved. At the same time, sequence *S*_5_ containing column numbers {1, 2, …, *K′*} of the *WT_A_*_’_ matrix repeated *N* times was created. Then, we determined the sum of *F*_1_ = *F*_1_ + *WT*(*s*_5_(*i*),*n*), where *n* = *s*_4_(*i* − 1) + (*s*_4_(*i*) − 1) × 4 was calculated for all *i* from 2 to *L* = *K’N*, for which *s*_4_(*i* − 1) and *s*_4_(*i*) were not gaps, whereas for those *i* for which *s*_4_(*i* − 1) was a gap, the sum was calculated as *F*_2_ = *F*_2_ + *E*(*s*_5_(*i*),*s*_4_(*i*)). Matrix *E* was calculated from the *WT_A_*_’_ matrix using Formula (6). We also calculated *F*_3_ = −*k*_1_*del*, where *k*_1_ was the number of gaps in alignment *A*’, and *del* was the insertion/deletion penalty (Formula (5)), as well as *F*_4_ = −*k*_2_*del*, where *k*_2_ was the difference in the number of nucleotides between alignments *A* and *A*’. Finally, we calculated *F*(*KN*’, *KN*’) = *F*_5_ = *F*_1_ + *F*_2_ − *F*_3_ − *F*_4_.

Weight matrix *WT_A_*_’_ is the image of alignment *A*’, for which statistical significance can be estimated based on the effectiveness of the alignment between the *WT_A_*_’_ matrix and random sequences. If the alignment is random, then matrix *WT_A_*_’_ would be random too and *F*_5_ would be close to the value obtained for random sequences (Section 2.2). 

Then, sequence *S*_4_ was randomly shuffled to create 200 sequences and matrix *WT_A_*_’_ was included in the *Q* set as described in Section 2.5. Each of the 200 sequences were treated as described in Section 2.2, Section 2.3, Section 2.4. As a result, 200 *maxV*(*n*_1_), each for a different random sequence, were obtained and used to calculate the mean *maxV(n_1_)* and variance $\sqrt{D(\max V(n_{1}}))$. Then, we calculated *Z* using Formula (7), where *F*_5_ was used rather than *maxV(n_1_)*. The MSA constructed by different mathematical methods, including MAHDS, had the same algorithm for calculating *Z*, which allowed their comparison based on *Z* values (supplementary material 1).

### 2.8. Algorithm for the Classification of Promoter Sequences from the A. thaliana Genome

The MAHDS algorithm developed in this study was applied to align promoter sequences from the *A. thaliana* genome (downloaded from https://epd.epfl.ch//index.php [33]). Each promoter had length *K* (600 nt), which included the region from −499 to +100 bp relative to the first base of the start codon (position +1). There were 22,694 promoter sequences in the analyzed set denoted as *PM* (supplementary material 1). Since the algorithm shown in Figure 1 requires considerable resources to align all the promoter sequences, we created a sample containing 500 randomly chosen promoters, which were combined into one sequence *S* with *L* = 500 × 600 = 30,000 nt. Then, we constructed the MSA as described in Figure 1 and Section 2.1, Section 2.2, Section 2.3, Section 2.4, Section 2.5, Section 2.6 and obtained *mV*(*n*_1_), two-dimensional alignment of sequences *S*_1_ and *S*, and PWM *mW*(600, 16). 

However, the volume of the *PM* set was significantly larger than the 500 randomly selected promoters included in sequence *S*. Furthermore, promoter sequences from the *PM* set might not show statistically significant alignment with *maxW*(600, 16). Therefore, we aligned each promoter from the *PM* set with matrix *maxW*(600, 16) using Formula (5) and considering the promoter sequence as *S* with *L* = 600. As a result, *F*(*L*, *L*) for each promoter from the *PM* set was calculated and put into the *Ves*(*i*) vector (where *i* is the promoter number).

Then, the promoter sequences with statistically significant *Ves*(*i)* were selected from the *PM* set. To do this, we used *PMR*(*i*) sets obtained by random shuffling of the promoter sequence with number *i*; each *PMR*(*i*) set contained 10^3^ random sequences of 600 bp. We aligned each sequence from *PMR*(*i*) relative to the *maxW*(600, 16) matrix, calculated *F*(*L*, *L*) denoted as *Vesr*(*j*) (*j* = 1, 2, …, 10^3^), and then determined the mean *Ves*(*j)* and variance *D*(*Vesr*) and calculated *Z* for each *Ves*(*i*) using Formula (7). If *Z* > *Z*_0_, then the promoter was considered to have a statistically significant alignment with the *maxW*(600, 16) matrix. For *Z*_0_ = 5.0, the probability of random similarity between the promoter and *maxW*(600, 16) was about 10^−6^. All promoter sequences with Z > 5.0 were assigned to the same class characterized by the *maxW*(600, 16) matrix.

When we created the first class of the *A.thaliana* promoter sequences in this way, we removed all the sequences with Z > 5.0 from the *PM* set and created *PM*(1) set. The resulting set *PM*(1) was used to create further classes. The described procedure was repeated for the *PM*(1) set, from the creation of a new set of 500 randomly selected promoters. As a result, we created a second class of promoters and a *PM*(2) set. We repeated this procedure for the sets *PM*(*i*), *i* = 1,2, …. Each iteration created a new class and the corresponding *maxW*(600, 16) matrix. If on some iteration, the volume of the PM(*i*) set became less than 500 sequences, then we chose all the sequences for carrying out the multiple alignment. The multiple alignments generated for each class are shown in Supplementary material 1. The procedure was stopped at the iteration *i* = *i*_0_ when the size of classes with *i* > *i*_0_ was less than 100 sequences. We defined the size of classes equal to 100 based on the random sequence analysis. When we performed the procedure on randomly shuffled promoter sequences (total number is 22,694), the volume of the classes ranged from 6 to 27 sequences with an average value of 16 sequences. This means that with using the threshold we kept the type I error rate less than 16%.

### 2.9. Divergence of Dinucleotide Positions in the Created Promoter Classes

We examined the difference in dinucleotide frequencies among the constructed MSAs based on the expected frequencies for each of the 599 positions (from −499 to +100) in the promoter regions. For this, we used the MSA obtained for sequence *S* in each class (see Section 2.8). Formula (1) was used to fill in the *M^k^ (600,16)* matrix, where *k* is the class number from 1 to 25 (see Section 3.2). The frequencies of nucleotides *f*(*j*) (*j* = 1, 2, 3, 4 for a, t, c, and g, respectively) and probabilities *p*(*j*) = *f*(*j*)/(*f*(1) + *f*(2) + *f*(3) + *f*(4)) were calculated for *S*. Then, we calculated the expected dinucleotide frequencies as *t*(*i*, *j*) = *Np*(*i*)*p*(*j*), where *N* is the number of promoters in the constructed MSA, base *i* is observed at position *l* − 1, and base *j* is observed at position *l* in the MSA with class *k*. Finally, we calculated variance *D*(*i*, *j*) *= Np*(*i*)*p*(*j*)(1 *− p*(*i*)*p*(*j*) and then *Z^k^(l, n) = (Mk(l, n) − t(i, j))/D(i, j)* for each element of the *M^k^(l,n)* matrix, where *n* ranged from 1 to 16 (*n* = *i* + 4(*j* − 1)) and *l*—from 2 to 600. Therefore, we could access the difference between the frequency of a base pair (*i, j*) at position *l* in the MSA and the expected frequency. *Z^k^(l, n)* is the normal approximation for the binomial distribution. The larger *Z^k^(l, n)*, the greater the difference between the observed and expected frequencies. 

To estimate the conservation of position *l*, we used sum $\chi^{k}(l)=\sum_{n=1}^{16}{\left(Z^{k}(l,n)\right)}^{2}$, which follows the *χ^2^* distribution with 15 degrees of freedom. We transformed *χ^k^(l)* into an argument of normal distribution using the normal approximation for chi-squared distribution $X^{k}(l)=\sqrt{2\chi^{k}(l)}-\sqrt{2f-1}$, where the number of degrees of freedom (*f*) is 15. As a result, function *χ^k^(l)* was obtained for multiple alignment *k*, where *l* ranges from 2 to 600. The greater *χ^k^(l)*, the more conserved is position *l* in the MSA.

## 3. Results

### 3.1. Comparison of MSA Methods Using Artificial Sequences

To compare different MSA methods, we generated *G*(*x*) sets, where each *G*(*x*) contained 100 sequences 600 nt long and *x* was the average number of substitutions per nucleotide (ranging from 0 to 4.0) for each pair of sequences from the *G*(*x*) set. In each *G*(*x*) set, we made 25 insertions and 25 deletions in random positions of randomly selected sequences (the number of introduced indels was based on the average number of indels found in the multiple alignment of promoter sequences performed in Section 3.2). We estimated the dependence of statistical significance (*Z*) on *x* for the following MSA methods: ClustalW [27] (https://www.genome.jp/tools-bin/clustalw), Clustal Omega [28], T-Coffee [17], Kalign [29], MAFFT [14], and Muscle [18] (https://www.ebi.ac.uk/Tools/msa/). The alignment was performed with the highest possible gap penalty.

**Figure 4:**
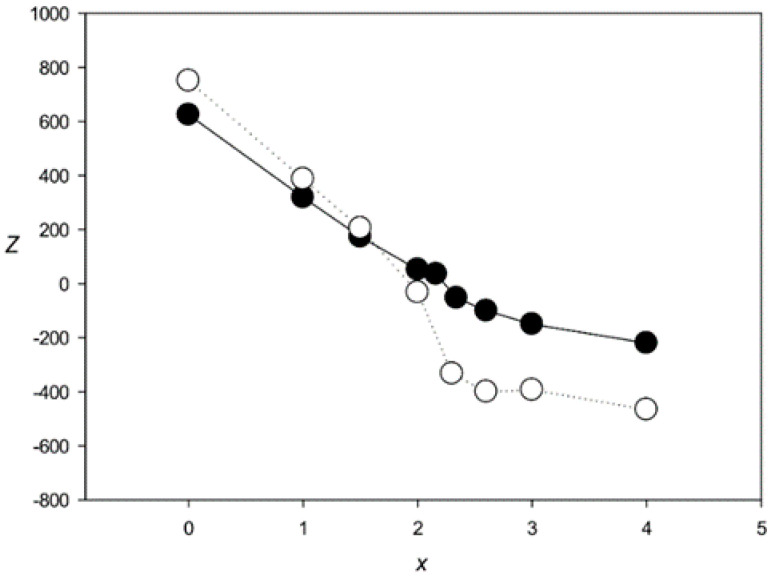
*Z*(*x*) for MSAs produced by ClustalW [27] (black circles) and Clustal Omega [28] (white circles).

**Figure 5:**
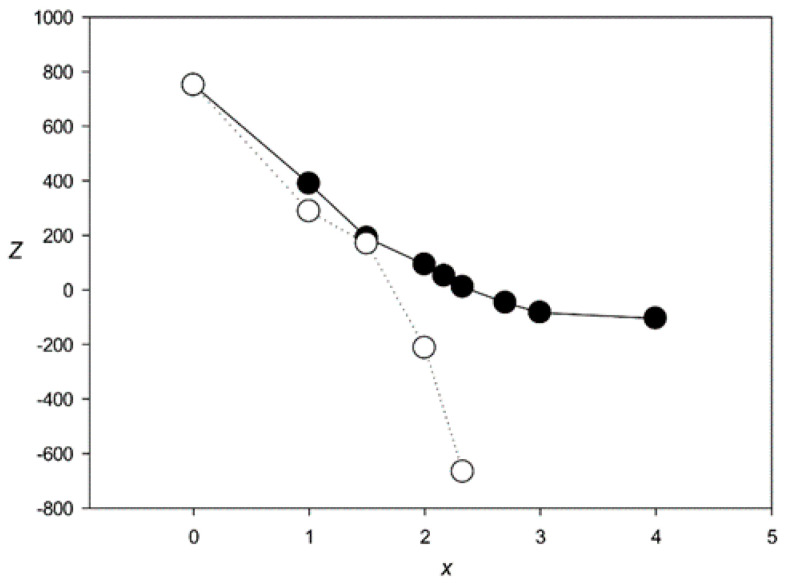
*Z*(*x*) for MSAs produced by MAFFT [14] (black circles) and T-Coffee [17] (white circles).

**Figure 6:**
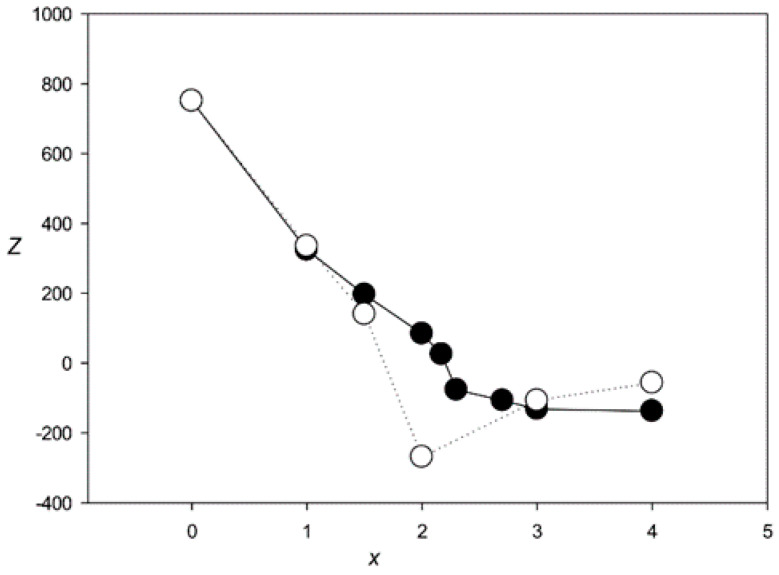
*Z*(*x*) for MSAs produced by Muscle [18] (black circles) and Kalign [29] (white circles).

Figure 4 shows the *Z*(*x*) function for ClustalW and Clustal Omega. The results indicated that both algorithms created statistically insignificant alignments (*Z* < 5.0) for sets with *x* > 2.1 and produced a very large number of indels (over 6000) at *x* > 2.0. The same situation was observed for the other tested algorithms—MAFFT, T-Coffee, Kalign, and Muscle (Figure 5 and Figure 6), which produced statistically insignificant alignments with *x* > 1.7, 2.5, 1.6, and 2.2, respectively.

Next, we evaluated the quality of MSAs created by these methods based on the coincidence of the total number of indels in the MSA with those in model sequences. The results showed that at *x* > 0.5, the number of indels exceeded 50 and at *x* > 1.0, this number could be several hundred, indicating that the quality of alignments produced by these methods is not high.

We also evaluated PRRN [19], MAVID [34], FSA [35], and CHAOS/DIALIGN [36]. However, for all these algorithms, it was not possible to obtain a statistically significant alignment for the sets with *x* > 2.5.

**Figure 7:**
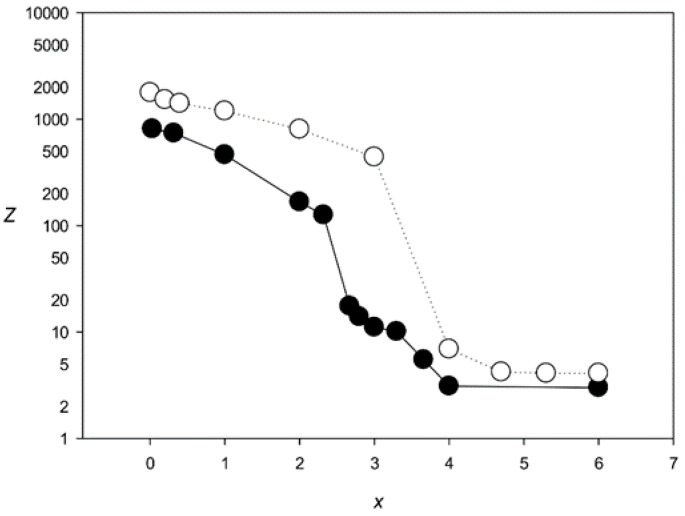
*Z*(*x*) for MSAs produced by the MAHDS algorithm. Black circles, 100 sequences with 50 indels; white circles, 500 sequences with 250 indels.

The results obtained with the MAHDS algorithm developed in this work are shown in Figure 7. MAHDS could produce statistically significant MSAs at *x* < 3.7, indicating that our method can build MSA for more diverged sequences than the most successful program T-Coffee (*x* < 2.5). Furthermore, the mean number of indels was about 48 for all *x* < 3.7, which is very close to the original number of indels (50) we introduced in each *G*(*x*) set.

We also applied MAHDS for *G*(*x*) sets containing 500 sequences with 250 indels (this number was based on the results of aligning promoter sequences in Section 3.3). In this case, MAHDS created statistically significant MSAs for *x* < 4.4 (Figure 7) and the total number of indels in the constructed alignments was around 254 for all *x* < 4.4, indicating that the increase in the number of analyzed sequences allows creating significant alignment for more divergent sequences (higher *x*).

The performance of MAHDS was also tested on the set of AluY non-coding transposable repeats abundant in the human genome. The AluY subfamily comprises sequences with an average length of 311 bp and various degrees of similarity [37]. We selected repeats from the AluY subfamily, which had at least 100 Alu elements with same degrees of identity (*ID*), and created *Al*(*ID*) sets. For each of the eight *ID* intervals (Table 2), there was one sequence, which had the identity with all the others in a given interval and this sequence was not included in the *Al* set. MSAs were constructed using ClustalW and MAHDS and their statistical significance was calculated following the same procedure as for the model sequences (Figure 4 and Figure 7). The results indicated that statistical significance decreased with *ID* and that ClustalW could not build statistically significant MSAs for *IDs* 0.4 ± 0.04 and 0.32 ± 0.04, whereas MAHDS could (Table 2). For *IDs* below 0.32 ± 0.04, it was not possible to construct an *Al* set since there were no repeats with this level of similarity.

A rough conversion of *ID* to *x* (*x* = 2 × (1.0 − *ID*)) is possible only for *ID*s over 0.45. In the case of lower *ID*s, we are in the so-called twilling zone, where there is no unambiguous relationship between *ID* and *x* and it is not possible to convert *ID* to *x*, since pairs of nucleotide sequences can have the same identity in a wide range of substitution numbers per nucleotide. For amino acid and nucleotide sequences, the thresholds are over 25% [38] and 40% [39], respectively. Therefore, for real DNA sequences, it is always possible to calculate *ID* but not *x* if *ID* is below 40%, since the real number of nucleotide substitutions is unknown. Therefore, *x* can be estimated only for model sequences. We could only conclude that at *ID* > 0.45 (*x* ≈ 1.1), MAHDS could produce statistically significant MSAs, but ClustalW could not. The decrease in the *x* threshold from 2.1 (model sequences, Figure 4) to 1.1 (Alu repeats), which is observed for ClustalW, is due to the decrease in the sequence length from 600 to 311 nt.

### 3.2. Creating classes for Promoter Sequences from the A. thaliana Genome

The iterative classification algorithm for promoter sequences described in Section 2.8 was used to calculate *maxW*(600, 16) for each class of *A. thaliana* promoters. The iterative algorithm stopped at *i* = 25, since the class size for *i* > 25 was less than 100 sequences. If such a procedure was performed for purely random sequences, the class size ranged from 6 to 27 sequences (16 in average), indicating that the promoter classification was performed with the type I error rate not exceeding 16%. Only the 18th and 24th classes deviated from this pattern, since their sets contained 65 and 47 sequences. We did not stop the classification procedure on these sets, since the size of the subsequent sets was more than 100 sequences.

**Figure 8:**
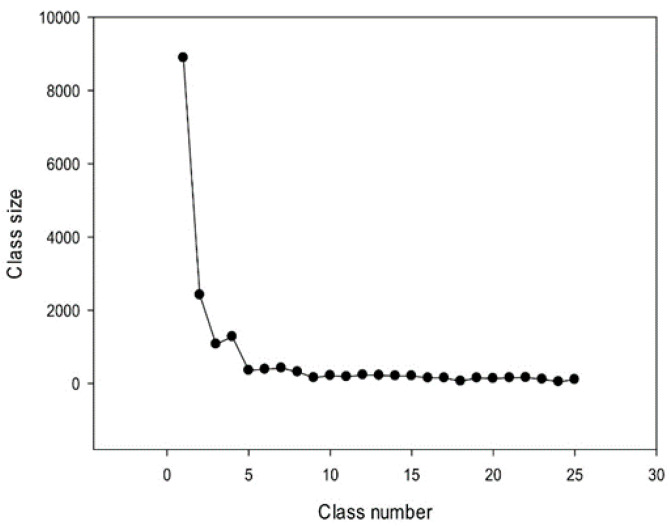
Sizes of 25 classes of promoter sequences identified in the *A. thaliana* genome.

Consequently, we obtained 25 classes of promoter sequences from the *A. thaliana* genome. The class distribution of promoter sequences is shown in Figure 8. The largest class contained more than 8888 promoters, followed by classes with 2419, 1071, and 1275 sequences. Classes from the 5th to 8th contained 300–400 promoters, and the smallest (25th) class contained 102 sequences. In total, 25 classes comprised 17,787 sequences constituting over 78% of all promoter sequences (22,703) present in the *A. thaliana* genome.

The number of classes strongly depended on the threshold value *Z*_0_ (Section 2.8). If *Z*_0_ increased, the number of classes for the 17,787 promoters was also increased and the number of promoters in each class was consequently decreased. At the same time, the size of the class obtained for random sequences (Section 2.8) was significantly reduced at *Z*_0_ > 6.0 (to 1–9 sequences). This classification can be performed for any group of sequences. The resulting MSAs for each class are shown in the appendix.

### 3.3. Conserved Positions in the Created Promoter Classes

**Figure 9:**
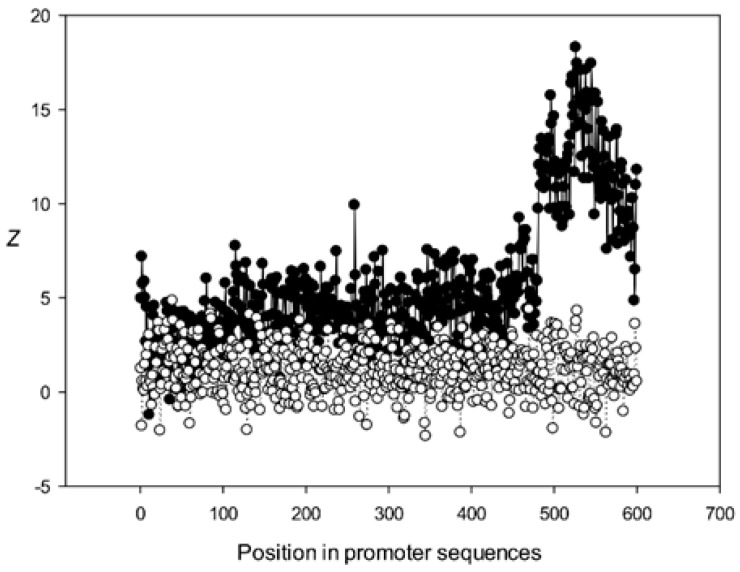
Dependence of *Z* on position *l* for promoter sequences of the first class. Black circles indicate multiple alignment of promoter sequences of the first class and white circles indicate randomly mixed promoter sequences where positions of indels were maintained.

**Figure 10:**
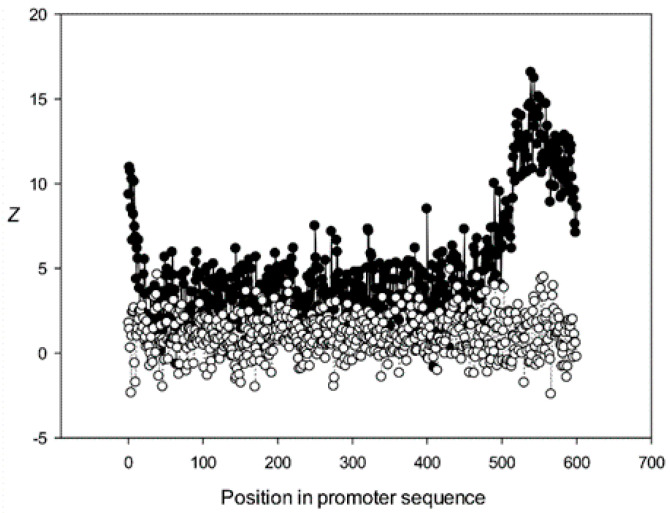
Dependence of *Z* on position *l* for promoter sequences of the second class. Black circles indicate multiple alignment for promoter sequences of the second class and white circles indicate randomly mixed promoter sequences where positions of indels were maintained.

**Figure 11:**
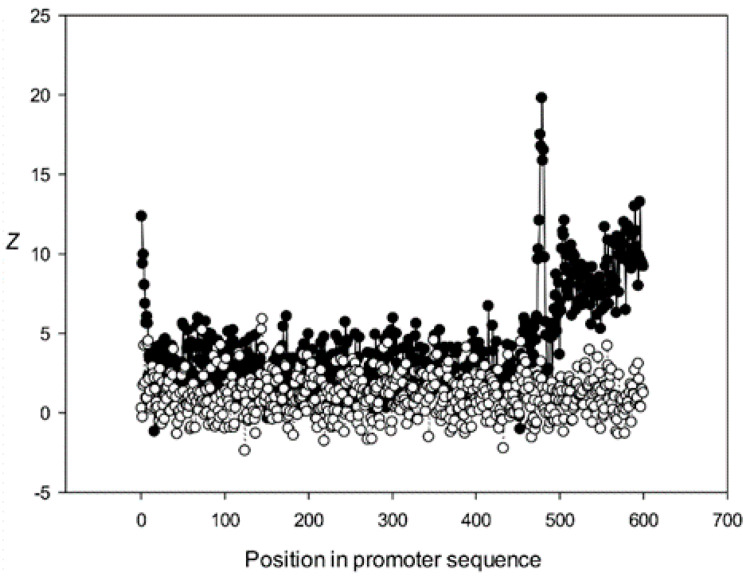
Dependence of *Z* on position *l* for promoter sequences of the third class. Black circles indicate multiple alignment for promoter sequences of the third class and white circles indicate randomly mixed promoter sequences where positions of indels were maintained.

**Figure 12:**
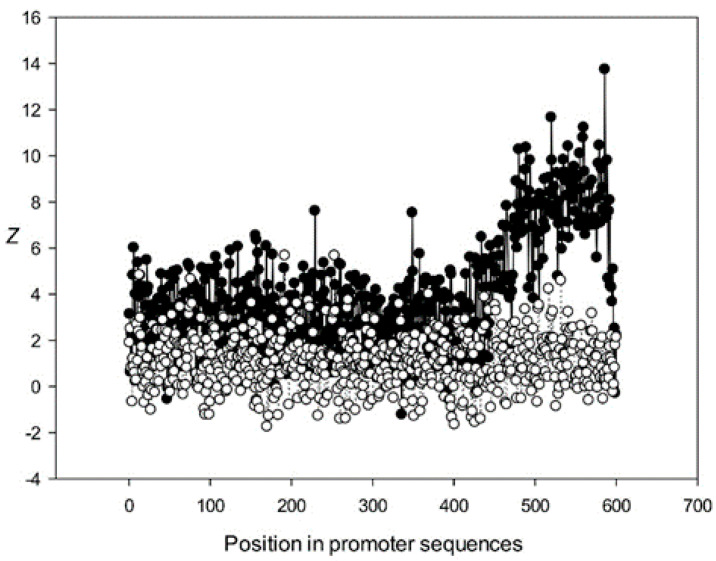
Dependence of *Z* on position *l* for promoter sequences of the fourth class. Black circles indicate multiple alignment for promoter sequences of the fourth class and white circles indicate randomly mixed promoter sequences where positions of indels were maintained.

**Figure 13:**
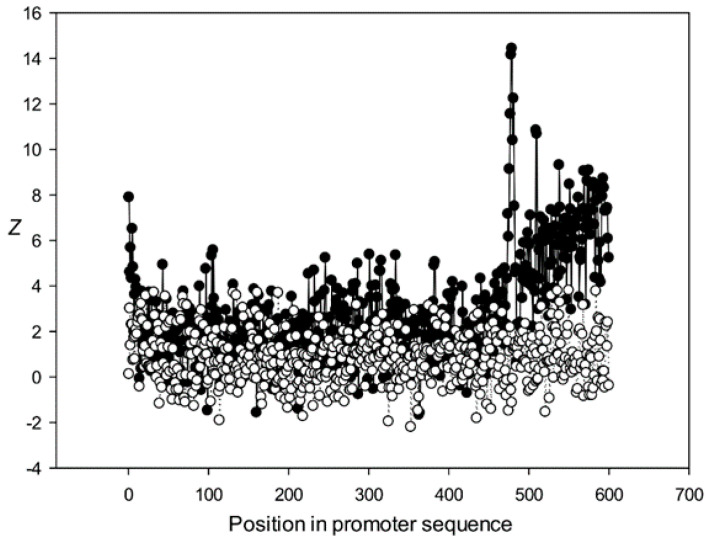
Dependence of *Z* on position *l* for promoter sequences of the fifth class. Black circles indicate multiple alignment for promoter sequences of the fifth class and white circles indicate randomly mixed promoter sequences where positions of indels were maintained.

Next, we analyzed the conservation of dinucleotides in the *A. thaliana* promoters based on the constructed MSAs. For this, we calculated *χ^k^(l)*, where *k* is the class number (Section 2.9). The graphs for the first five classes (*k* 1–5) are shown in Figure 9, Figure 10, Figure 11, Figure 12 and Figure 13. The results for the first class containing 8888 sequences indicated that conserved positions were present almost everywhere along the promoter sequences and that the dinucleotide frequencies were significantly different from the expected frequencies calculated for random sequences (Figure 9). 

The region from +1 to +80 (501–580 nt in Figure 9) was the most highly conserved in the first class and a similar phenomenon was also observed in all other classes. It has been previously shown that there is a promoter element (DPE) located 28–33 nt downstream from the start codon [40,41,42], which is widely distributed among promoter sequences and is similar to the TATA box [43,44]. However, only relatively short conserved sequences (about 7 bases) elements are observed in promoter sequences. It can be suggested that the expression of various proteins may depend on the downstream 1–80-bp region, which is necessary for transcription initiation and may also play a role in the other processes. The peak at position 501 corresponded to the first codon (+1) and that at position 470—to the TATA-box (−30) in the first class (Figure 9). The first codon was also well marked in the third class (Figure 11) and the TATA box was best identified in the third and fifth classes (Figure 11 and Figure 13), but could also be seen in the other classes (Figure 10 and Figure 12).

Conservation was also found in the region upstream of the start codon (from −499 to –30 bp corresponding to 1–470 bp of the analyzed sequences), suggesting similarities in transcriptional mechanisms for the corresponding genes. The dinucleotide frequencies in this region differed from the expected frequencies calculated for random sequences. It is somewhat surprising that even sufficiently distant positions (the first base in Figure 13) can be conserved.

We also analyzed the statistical significance of multiple alignment of promoter sequences. MSAs for the first four classes with the largest numbers of promoters contained 220–280 indels. The classes were initially created by aligning 500 promoters (Section 2.8). To estimate the degree of divergence among the promoter sequences, we conducted multiple alignments for 500 artificial sequences containing 250 indels and different numbers of substitutions per nucleotide (Figure 7), calculated *F*(*L*, *L*) for the simulated sequences (which was equal to *F*(*L*, *L*) of classes 1–4), and determined the corresponding *x* values showing the average number of substitutions per position. The results indicated that MSAs for classes 1–4 had *x* = 3.7.

The alignments of the promoter sequences from classes 1–4 created using MAFFT, T-Coffee, Kalign, Muscle, ClustalW, and Clustal-Omega programs were not statistically significant (*Z* < −2.0) and contained a very large number of indels. Since according to our estimations, the promoter sequences of these classes carried approximately 3.7 mutations per nucleotide, the alignment cannot be calculated by these methods.

## 4. Discussion

In this work, we developed the MAHDS algorithm for MSA and created a server for its application available at http://victoria.biengi.ac.ru/mahds/main. MAHDS can perform the alignment for nucleotide sequences with a high degree of diversity, which cannot be done with the other method, including ClustalW [27], Clustal-Omega [28], T-Coffee [17], Kalign [29], and MAFFT, which align sequences that accumulated no more than 2.5 substitutions per nucleotide (*x* < 2.5). In this study, we used default parameters, however, we also tried other settings. Therefore, we examined the statistical significance of aligning model sequences by ClustalW using eight different values of gap open (Go) penalties in the interval from 2 to 30 and gap extension (Ge) penalties from 0.1 to 25% for each Go value. For *Z* = 5.0, the maximum *x* was 2.4 and in default conditions, *x* was 2.1 (Figure 4), which means that variations in Go/Ge penalties can increase *x* by ~15%, which is within the error shown in Figure 4. To estimate the error in calculating *Z*, we used different *G*(*x*) sets (Section 3.1) generated from different initial sequences. It is quite unlikely that a heuristic algorithm ClustalW that progressively builds MSA from a series of pairwise alignments could produce results with *Z* > 5.0 for *x* > 2.5. Replacing the PAM matrix with BLOSUM did not significantly affect the results shown in Figure 4 and all changes were within the error shown there. For the other methods used in Section 3.1, the increase in *x* also did not exceed ~15%.

In contrast to the other algorithms, MAHDS calculates statistically significant MSA for *x* < 3.7; furthermore, if the number of sequences to be aligned increases to 500, then the limit for *x* increases to < 4.4. We believe that such a capability of MAHDS is very important for MSA of different genes and regulatory sequences and could provide higher precision in their annotation. To align 100 sequences of 600 nt each by the MAHDS method, we used a computer cluster with 64 computing cores (eight Ryzen 7 1700 processors) and the alignment took less than 6 min.

It is usually a challenge to perform the alignment of promoter sequences due to their considerable dissimilarity [45]. Despite the large number of promoters, it has not been possible to obtain a statistically significant multiple alignment of their sequences [46]. By using MAHDS, we could construct a statistically significant multiple alignment for ~ 78% of the known promoter sequences from the *A. thaliana* genome. The estimated average number of substitutions per nucleotide (*x*) in the created promoter classes was 3.7, whereas the other methods provided statistically significant MSAs at *x* < 2.5, which explains why such MSA of promoters has not been obtained previously. Therefore, MAHDS could perform multiple alignment of sequences with a low degree of similarity, i.e., those that have accumulated a considerable number (3.7) of substitutions per nucleotide, which enabled us to classify promoters and reveal some common properties within each class at a statistically significant level. However, the developed MAHDS algorithm, similar to the existing methods for computing MSA (MAFFT, T-Coffee, Kalign, Muscle, ClustalW, and Clustal-Omega), is purely mathematical and, therefore, can only reveal structural similarities among the aligned sequences but not their biological significance. At this point, it is difficult to conclude whether the revealed patterns are inherent to promoters or are also present in other sequences. Similarly, it is unclear whether the observed sequence similarity is associated with a common evolutionary origin of promoter regions or with the general functional role of promoters in gene transcription. These problems should be addressed in special studies, where the promoter classes and MSAs obtained here would be used to identify promoter sequences in various genomes and correlate them with experimental results, which can be done using HMMs [47,48]. 

The search for potential promoters in various genomes is a major challenge. The currently available predictive algorithms such as TSSW [45], PePPER [49], and G4PromFinder [50] use the existing mathematical methods, which do not provide a statistically significant alignment of diverse sequences. The best algorithms can predict one false positive point in 10^3^–10^4^ DNA bases. As a result, it is not possible to distinguish the true promoter from false hits. For the promoter prediction, it will be convenient to use *maxW*(600, 16) matrices calculated here and the mathematical algorithm described in Section 2.3 as various successively cut genome fragments can be considered as sequence *S.* We believe that a number of false positive hits will be several hundred or even a few dozen per genome size of 3 × 10^9^ bases.

In this work, we used MAHDS to construct the multiple alignment of promoter sequences. The constructed alignment can be used for the subsequent prediction of promoters in genome sequences and to improve the accuracy of these predictions. In this case, to reduce the number of false positives it is important to get MSA with the highest statistical significance (as we wrote above). However, if MAHDS is used for other purposes, it can be important to estimate the biological correctness of the constructed MSA. Methods developed for the evaluation of MSA accuracy and correctness utilize structural information about corresponding proteins [51]. However, here we consider DNA sequences for which, according to our estimate, *x* ~ 3.7. To our knowledge, there are no biologically correct MSA constructed for the set of all promoter sequences. As well as there are no such MSA for any DNA sequences with *x* ~ 3.7. The point is that both pairwise and multiple alignments could be constructed only for sequences with *x* < 2.5, as shown in Figure 4, Figure 5 and Figure 6. Therefore, in this work, we focused on the statistical significance of the constructed alignments, which is a common statistical technique. We believe that a biologically correct alignment using MAHDS for *x* > 2.5 can be constructed in the future if one can find amino acids or DNA sequences with a similar structure but with sequences’ *x* ~ 3.5–3.7. Then, it will be possible to improve the accuracy of the MAHDS algorithm in the same way as it was done for other methods [51]. However, even the present form of MAHDS can be used to predict promoter sequences, as we wrote in the paragraph above.

In our future research, we will focus on the development of the software for protein MSA based on the same method. For protein sequences, the number of possible amino acid pairs is 400 and a large number of sequences (over 2000) would be required to fill in the *maxW*(*L*/*N*, 400) matrix, where *L* is the total sequence length, *N* is the number of sequences, and *L*/*N* is the average length of sequences. Therefore, it will be problematic to use this approach for small samples (less than 100 sequences), when the statistically significant filling of the *maxW*(*L*/*N*, 400) matrix is not possible. We intend to apply a series of optimization procedures to address this issue. 

## 5. Conclusions

In this study, we applied a new mathematical method (MAHDS) for performing multiple alignment of highly divergent sequences, i.e., sequences that have in average more than 2.5 substitutions per position (*x*). The results indicated that most of the existing methods could produce statistically significant alignments only for the sets with *x* < 2.5, whereas MAHDS could operate on sequences with *x* = 4.4. We have created a web server for multiple alignment of nucleotide sequences located at http://victoria.biengi.ac.ru/mahds/main. Then we performed multiple alignments of promoter sequences from the *A. thaliana* genome and created 25 classes of promoter sequences. Each class of promoter sequences has a statistically significant multiple alignment. The obtained multiple alignments can be used to improve methods for searching for promoter sequences in a variety of genomes.

## Figures and Tables

**Table 1:** An example of matrix transformation to the given *R*^2^ and *K_d_*.

Random Matrix *						Transformed Matrix **				
	1	2	3	4	5	1	2	3	4	5
aa	−1.6	−1.0	−1.6	−1.6	−1.6	−5.4	−2.9	−5.4	−5.3	−5.3
at	−1.7	1.7	−1.7	−1.0	3.2	−5.7	7.6	−5.7	−3.0	13.4
ac	−1.3	0.2	−1.3	−1.3	3.5	−4.1	1.9	−4.1	−4.0	14.7
ag	−1.3	−1.3	−0.6	−1.3	−1.3	−4.4	−4.4	−1.4	−4.2	−4.2
ta	−1.0	−1.7	2.7	−1.0	−1.7	−3.2	−5.7	11.4	−3.0	−5.6
tt	1.5	−1.1	−0.5	2.1	−0.5	6.8	−3.5	−1.2	9.2	−1.0
tc	1.7	−1.3	−0.6	−0.5	−1.3	7.7	−4.3	−1.3	−1.2	−4.2
tg	0.0	−0.7	1.5	−0.6	−1.4	1.1	−1.7	6.8	−1.5	−4.5
ca	−1.3	−1.3	0.2	−1.3	−1.3	−4.1	−4.1	1.9	−4.0	−4.0
ct	0.1	−0.6	−1.3	1.7	−1.3	1.6	−1.3	−4.3	7.8	−4.2
cc	−1.0	−1.0	−1.0	0.9	−1.0	−3.1	−3.1	−3.1	4.5	−3.0
cg	−1.1	−1.1	−0.1	−1.0	−1.0	−3.3	−3.3	0.3	−3.2	−3.2
ga	−1.3	−1.3	−0.6	−1.3	−0.5	−4.4	−4.4	−1.4	−4.2	−1.2
gt	−0.7	3.0	−1.4	−1.4	−1.4	−1.7	12.5	−4.6	−4.5	−4.5
gc	−1.1	−1.1	−1.1	−1.0	−1.0	−3.3	−3.3	−3.3	−3.2	−3.2
gg	−1.1	−1.1	−1.1	−1.1	−1.1	−3.5	−3.5	−3.5	−3.4	−3.4

* *R*^2^ = 155; ** *R*^2^ = 2000 and K_d_ = −1.5.

**Table 2:** Calculation of the statistical significance for multiple alignments of Alu repeats from the human genome.

*Z*	*ID*							
	1.0	0.95 ± 0.01	0.84 ± 0.02	0.69 ± 0.03	0.52 ± 0.03	0.45 ± 0.4	0.4 ± 0.04	0.32 ± 0.04
*Z*_1_	486	382	362	297	171	130	−3.3	−22.3
*Z*_2_	579	394	366	256	152	119.0	29.0	22.1

*ID* is the degree of identity in the sets of Alu repeats; *Z*_1_ and *Z*_2_ are calculated for alignments created by ClustalW and MAHDS, respectively, using Formula (7).

## Supplementary Materials

The following are available online at https://www.mdpi.com/2073-4425/12/2/135/s1, Supplementary material 1: Data package.

## References

[1] Chatzou M., Magis C., Chang J.-M., Kemena C., Bussotti G., Erb I., Notredame C. (2016). Multiple sequence alignment modeling: Methods and applications. Brief. Bioinform..

[2] Morrison A.D., Russell D.J. (2015). Multiple sequence alignment methods.

[3] Blanchette M. (2007). Computation and analysis of genomic multi-sequence alignments. Annu. Rev. Genom. Hum. Genet..

[4] Elias I. (2006). Settling the intractability of multiple alignment. J. Comput. Biol..

[5] Chowdhury B., Garai G. (2017). A review on multiple sequence alignment from the perspective of genetic algorithm. Genomics.

[6] Wang L., Jiang T. (1994). On the complexity of multiple sequence alignment. J. Comput. Biol..

[7] Murata M., Richardson J.S., Sussman J.L. (1985). Simultaneous comparison of three protein sequences. Proc. Natl. Acad. Sci. USA.

[8] Hung C.-L., Lin Y.-S., Lin C.-Y. (2015). CUDA ClustalW: An efficient parallel algorithm for progressive multiple sequence alignment on Multi-GPUs. Comput. Biol. Chem..

[9] Waterman M.S., Jones R. (1990). Consensus methods for DNA and protein sequence alignment. Meth. Enzymol..

[10] Durbin R., Eddy S., Krogh A., Mitchison G. (1998). Biological Sequence Analysis: Probabilistic Models of Proteins and Nucleic Acids.

[11] Gonnet G.H., Korostensky C., Benner S. (2000). Evaluation measures of multiple sequence alignments. J. Comput. Biol..

[12] Thompson J.D., Higgins D.G., Gibson T.J. (1994). CLUSTAL W: Improving the sensitivity of progressive multiple sequence alignment through sequence weighting, position-specific gap penalties and weight matrix choice. Nucleic Acids Res..

[13] Thomsen R., Fogel G., Krink T. A Clustal alignment improver using evolutionary algorithms. Proceedings of the 2002 Congress on Evolutionary Computation. CEC’02 (Cat. No.02TH8600).

[14] Katoh K., Frith M.C. (2012). Adding unaligned sequences into an existing alignment using MAFFT and LAST. Bioinformatics.

[15] Katoh K., Rozewicki J., Yamada K.D. (2019). MAFFT online service: Multiple sequence alignment, interactive sequence choice and visualization. Brief. Bioinform..

[16] Simossis V.A., Kleinjung J., Heringa J. (2005). Homology-extended sequence alignment. Nucleic Acids Res..

[17] Notredame C., Higgins D.G., Heringa J. (2000). T-coffee: A novel method for fast and accurate multiple sequence alignment. J. Mol. Biol..

[18] Edgar R.C. (2004). MUSCLE: Multiple sequence alignment with high accuracy and high throughput. Nucleic Acids Res..

[19] Gotoh O. (1996). Significant improvement in accuracy of multiple protein sequence alignments by iterative refinement as assessed by reference to structural alignments. J. Mol. Biol..

[20] Brudno M., Chapman M., Göttgens B., Batzoglou S., Morgenstern B. (2003). Fast and sensitive multiple alignment of large genomic sequences. BMC Bioinform..

[21] Hughey R., Krogh A. (1996). Hidden Markov models for sequence analysis: Extension and analysis of the basic method. Bioinformatics.

[22] Grasso C., Lee C. (2004). Combining partial order alignment and progressive multiple sequence alignment increases alignment speed and scalability to very large alignment problems. Bioinformatics.

[23] Eddy S.R. Multiple Alignment Using Hidden Markov Models. Proceedings of the International Conference on Intelligent Systems for Molecular Biology.

[24] Baldi P., Chauvin Y., Hunkapiller T., McClure M.A. (1994). Hidden Markov models of biological primary sequence information. Proc. Natl. Acad. Sci. USA.

[25] Pugacheva V., Korotkov A., Korotkov E. (2016). Search of latent periodicity in amino acid sequences by means of genetic algorithm and dynamic programming. Stat. Appl. Genet. Mol. Biol..

[26] Korotkov E.V., Korotkova M.A. (2017). Search for regions with periodicity using the random position weight matrices in the C. elegans genome. Int. J. Data Min. Bioinform..

[27] Larkin M.A., Blackshields G., Brown N.P., Chenna R., Mcgettigan P.A., McWilliam H., Valentin F., Wallace I.M., Wilm A., Lopez R. (2007). Clustal W and Clustal X version 2.0. Bioinformatics.

[28] Sievers F., Wilm A., Dineen D., Gibson T.J., Karplus K., Li W., Lopez R., McWilliam H., Remmert M., Söding J. (2011). Fast, scalable generation of high-quality protein multiple sequence alignments using Clustal Omega. Mol. Syst. Biol..

[29] Lassmann T., Sonnhammer E. (2005). Kalign–An accurate and fast multiple sequence alignment algorithm. BMC Bioinform..

[30] Needleman S.B., Wunsch C.D. (1970). A general method applicable to the search for similarities in the amino acid sequence of two proteins. J. Mol. Biol..

[31] Laskin A., Korotkov E.V., Chaleĭ M.B., Kudriashov N. (2003). The locally optimal method of cyclic alignment to reveal latent periodicities in genetic texts. The NAD-binding protein sites. Мoлекулярная биoлoгия.

[32] Suvorova Y., Korotkova M., Skryabin K.G., Korotkov E.V. (2019). Search for potential reading frameshifts in CDS from Arabidopsis thaliana and other genomes. DNA Res..

[33] Dreos R., Ambrosini G., Groux R., Périer R.C., Bucher P. (2017). The eukaryotic promoter database in its 30th year: Focus on non-vertebrate organisms. Nucleic Acids Res..

[34] Bray N., Pachter L. (2004). MAVID: Constrained ancestral alignment of multiple sequences. Genome Res..

[35] Bradley R.K., Roberts A., Smoot M., Juvekar S., Do J., Dewey C., Holmes I., Pachter L. (2009). Fast statistical alignment. PLoS Comput. Biol..

[36] Brudno M., Steinkamp R., Morgenstern B. (2004). The CHAOS/DIALIGN WWW server for multiple alignment of genomic sequences. Nucleic Acids Res..

[37] Batzer M.A., Kilroy G.E., Richard P.E., Shaikh T.H., Desselle T.D., Hoppens C.L., Deininger P.L. (1990). Structure and variability of recently inserted Alu family members. Nucleic Acids Res..

[38] Chang G.S., Hong Y., Ko K.D., Bhardwaj G., Holmes E.C., Patterson R.L., Van Rossum D.B. (2008). Phylogenetic profiles reveal evolutionary relationships within the “twilight zone” of sequence similarity. Proc. Natl. Acad. Sci. USA.

[39] Mcgimpsey S. (2019). The Twilight Zone of Nucleotide Homology. Ph.D. Thesis.

[40] Burke T.W., Kadonaga J.T. (1996). Drosophila TFIID binds to a conserved downstream basal promoter element that is present in many TATA-box-deficient promoters. Genes Dev..

[41] Juven-Gershon T., Kadonaga J.T. (2010). Regulation of gene expression via the core promoter and the basal transcriptional machinery. Dev. Biol..

[42] Yang C., Bolotin E., Jiang T., Sladek F.M., Martinez E. (2007). Prevalence of the initiator over the TATA box in human and yeast genes and identification of DNA motifs enriched in human TATA-less core promoters. Gene.

[43] Kutach A.K., Kadonaga J.T. (2000). The downstream promoter element dpe appears to be as widely used as the tata box in drosophila core promoters. Mol. Cell. Biol..

[44] Kadonaga J.T. (2002). The DPE, a core promoter element for transcription by RNA polymerase II. Exp. Mol. Med..

[45] Solovyev V.V., Shahmuradov I.A., Salamov A. (2010). Identification of promoter regions and regulatory sites. Tox. Asses..

[46] Zeng J., Zhu S., Yan H. (2009). Towards accurate human promoter recognition: A review of currently used sequence features and classification methods. Brief. Bioinform..

[47] Claesen J., Burzykowski T. (2015). A hidden Markov-model for gene mapping based on whole-genome next generation sequencing data. Stat. Appl. Genet. Mol. Biol..

[48] Yoon B.-J. (2009). Hidden Markov models and their applications in biological sequence analysis. Curr. Genom..

[49] De Jong A., Pietersma H., Cordes M., Kuipers O.P., Kok J. (2012). PePPER: A webserver for prediction of prokaryote promoter elements and regulons. BMC Genom..

[50] Di Salvo M., Pinatel E.M., Talà A., Fondi M., Peano C., Alifano P. (2018). G4PromFinder: An algorithm for predicting transcription promoters in GC-rich bacterial genomes based on AT-rich elements and G-quadruplex motifs. BMC Bioinform..

[51] Kemena C., Notredame C. (2009). Upcoming challenges for multiple sequence alignment methods in the high-throughput era. Bioinformatics.

